# Supplementary material for: The Unique Lipidomic Signatures of Saccharina latissima Can Be Used to Pinpoint Their Geographic Origin
Source: Biomolecules. 2020 Jan 8;10(1):107. doi: 10.3390/biom10010107 (PMC7023228; doi:10.3390/biom10010107)
Supplement: Supplementary file 1 [file biomolecules-10-00107-s001.pdf]

Saccharina latissima from different culture locations display unique lipidomic signatures that can be used to trace their geographic origin

João P. Monteiro [1,2*], Felisa Rey[1,3], Tânia Melo[1,2], Ana Moreira[1,2], Jean-François Arbona[4], Jorunn Skjermo[5], Silje Forbord[5], Jon Funderud[6], Diogo Raposo[6], Philip D. Kerrison[7], Marie-Mathilde Perrineau[7], Claire Gachon[7], Pedro Domingues[1], Ricardo Calado[3] and M. Rosário Domingues[1,2]

[1] Centro de Espetrometria de Massa, Departamento de Química & QOPNA, Universidade de Aveiro, Campus Universitário de Santiago, 3810-193 Aveiro, Portugal

[2] Departamento de Química & CESAM & ECOMARE, Universidade de Aveiro, Campus Universitário de Santiago, 3810-193 Aveiro, Portugal

[3] Departamento de Biologia & CESAM & ECOMARE, Universidade de Aveiro, Campus Universitário de Santiago, 3810-193 Aveiro, Portugal

[4] C-Weed Aquaculture, 35400 St. Malo, France

[5] SINTEF Ocean, N-7465 Trondheim, Norway

[6] Seaweed Energy Solution AS, Bynesveien 48, 7018 Trondheim, Norway

[7] Scottish Association for Marine Science, PA37 1QA, Oban, UK

Supplementary Material

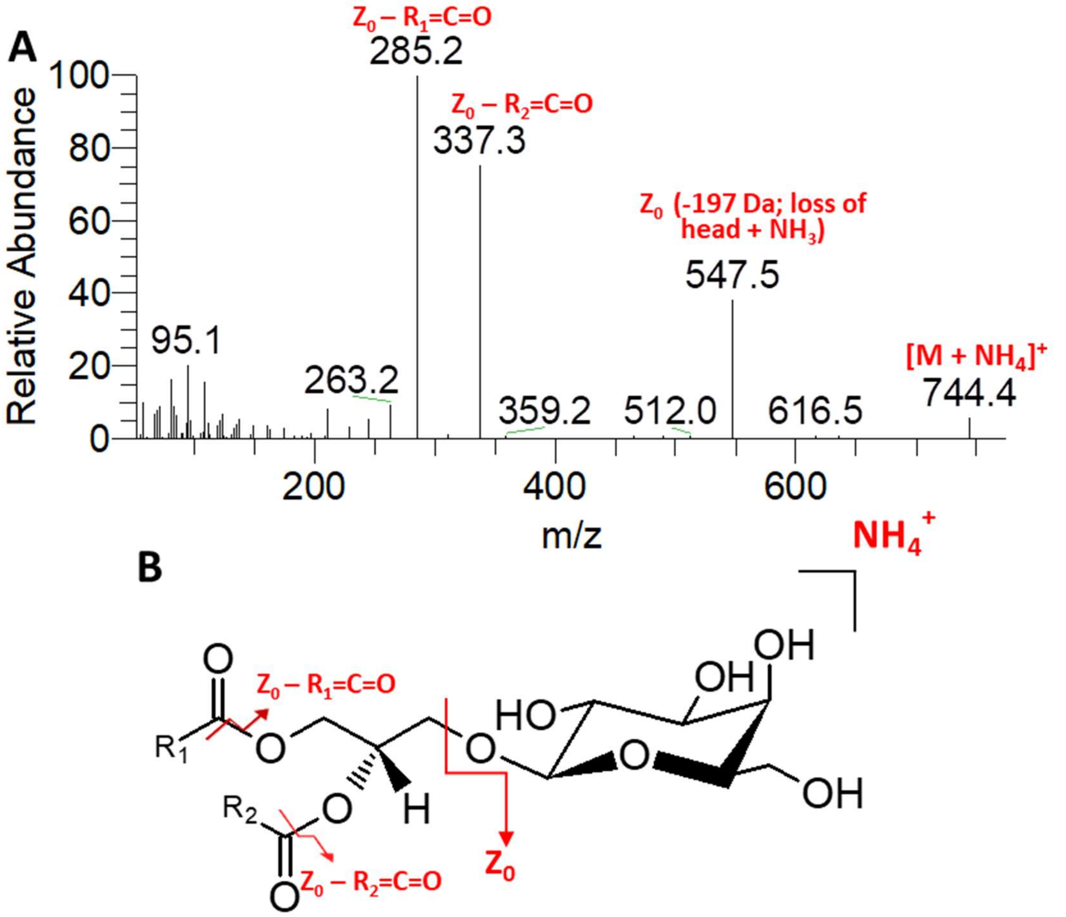

Supplementary Figure 1: Species of monogalactosyl diacylglycerol (MGDG) confirmation by MS/MS. A) HILIC-ESI-MS/MS spectrum of MGDG (32:2; 14:0/18:2) corresponding to $[M + NH_4]^+$ ion at *m/*z 744.6. B) Confirmation of galactolipid class was achieved by the identification of the neutral loss of hexose moiety and NH_3 (-197 Da), corresponding to the loss of the head group of MGDG. Fatty acid components were determined by identification of product ions formed by sequential neutral loss of hexose moiety and NH_3 followed by loss of fatty acyl chains as ketene (neutral loss of R=C=O). For MGMG confirmation the same principle is used, with the exception that only one loss of a fatty acid in ketene form is detected.

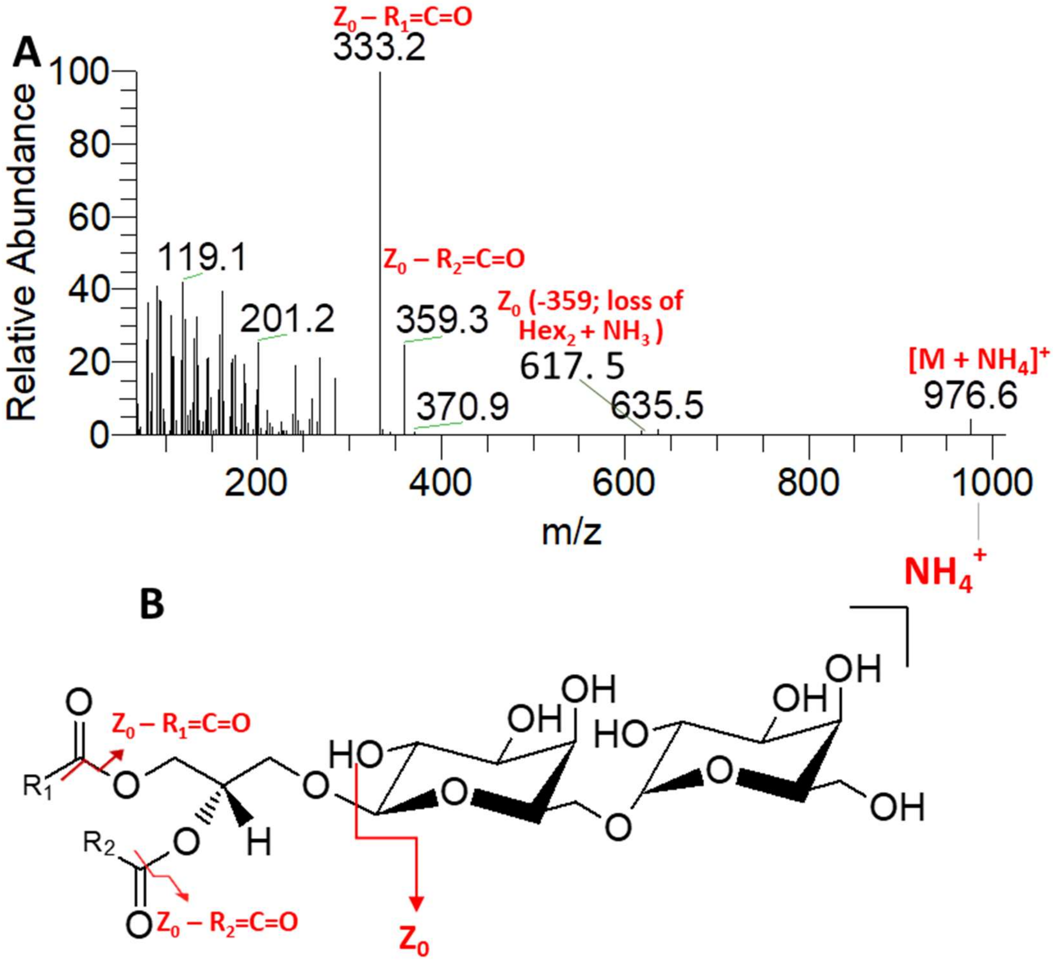

Supplementary Figure 2: Species of digalactosyl diacylglycerol (DGDG) confirmation by MS/MS. A) HILIC-ESI-MS/MS spectrum of DGDG (38:9; 18:4/20:5) corresponding to $[M + NH_4]^+$ ion at *m/*z 976.6. B) Confirmation of galactolipid class was achieved by the identification of the neutral loss of two hexose residues and H_20 (-359 Da), corresponding to the loss of the head group of DGDG. Fatty acid components were determined by identification of product ions formed by sequential neutral loss of hexose moiety and NH_3 followed by loss of fatty acyl chains as ketene (neutral loss of R=C=O). For DGMG confirmation the same principle is used, with the exception that only one loss of a fatty acid in ketene form is detected.

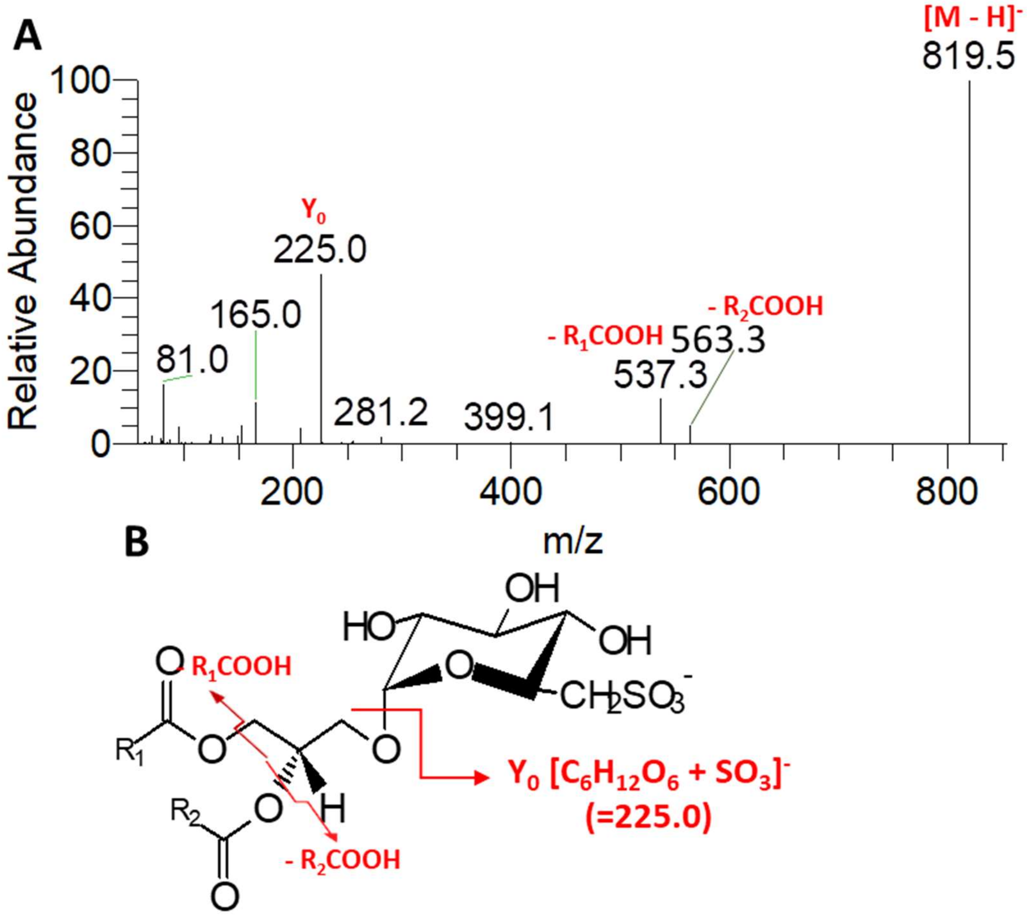

Supplementary Figure 3: Species of sulfoquinovosyl diacylglycerol (SQDG) confirmation by MS/MS. A) HILIC-ESI-MS/MS spectrum of SQDG (34:1; 16:0/18:1) corresponding to $[M - H]^-$ ion at *m/z* 819.5. B) Confirmation of sulpholipid class was achieved by the identification of the ion at *m/z* 225.0 corresponding to the sulfoquinovosyl group of the polar head of SQDG. Fatty acid components were confirmed by the identification of product ions corresponding to the neutral loss the fatty acyl chains (-RCOOH). For SQMG confirmation the same principle is used, with the exception that only one loss of a fatty acid acyl chain is detected.

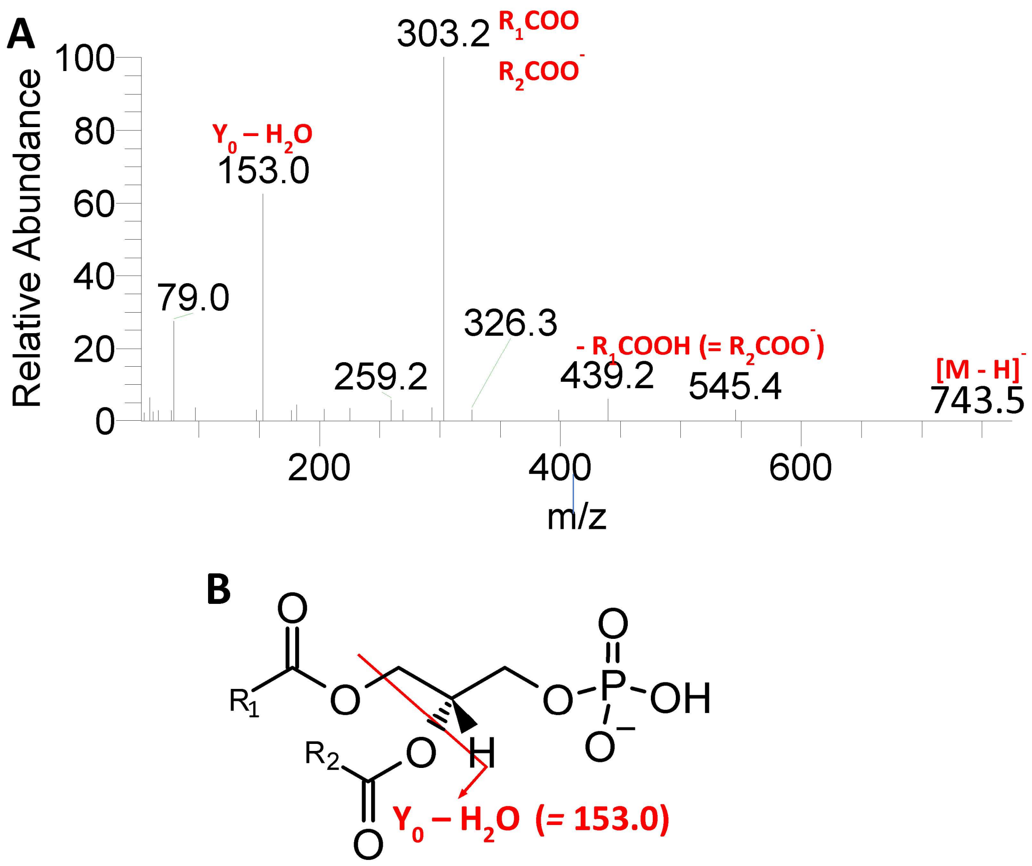

Supplementary Figure 4: Species of phosphatidic acid (PA) confirmation by MS/MS. A) HILIC-ESI/MS spectrum of PA (40:8; 20:4/20:4), corresponding to $[M - H]^-$ ion at *m/z* 743.5 in negative mode. B) Confirmation of phospholipid class was achieved by the identification of the ion at *m/z* 153.0, corresponding to glycerol phosphate anion -H_2O. Fatty acid components were confirmed by the identification of product ions corresponding to the fatty acyl chains as $[RCOO]^-$ or by the neutral loss of RCOOH or ketene forms of fatty acids R=C=O. For LPA confirmation the same principle is used, with the exception that only one product ion/loss corresponding to a fatty acid is detected.

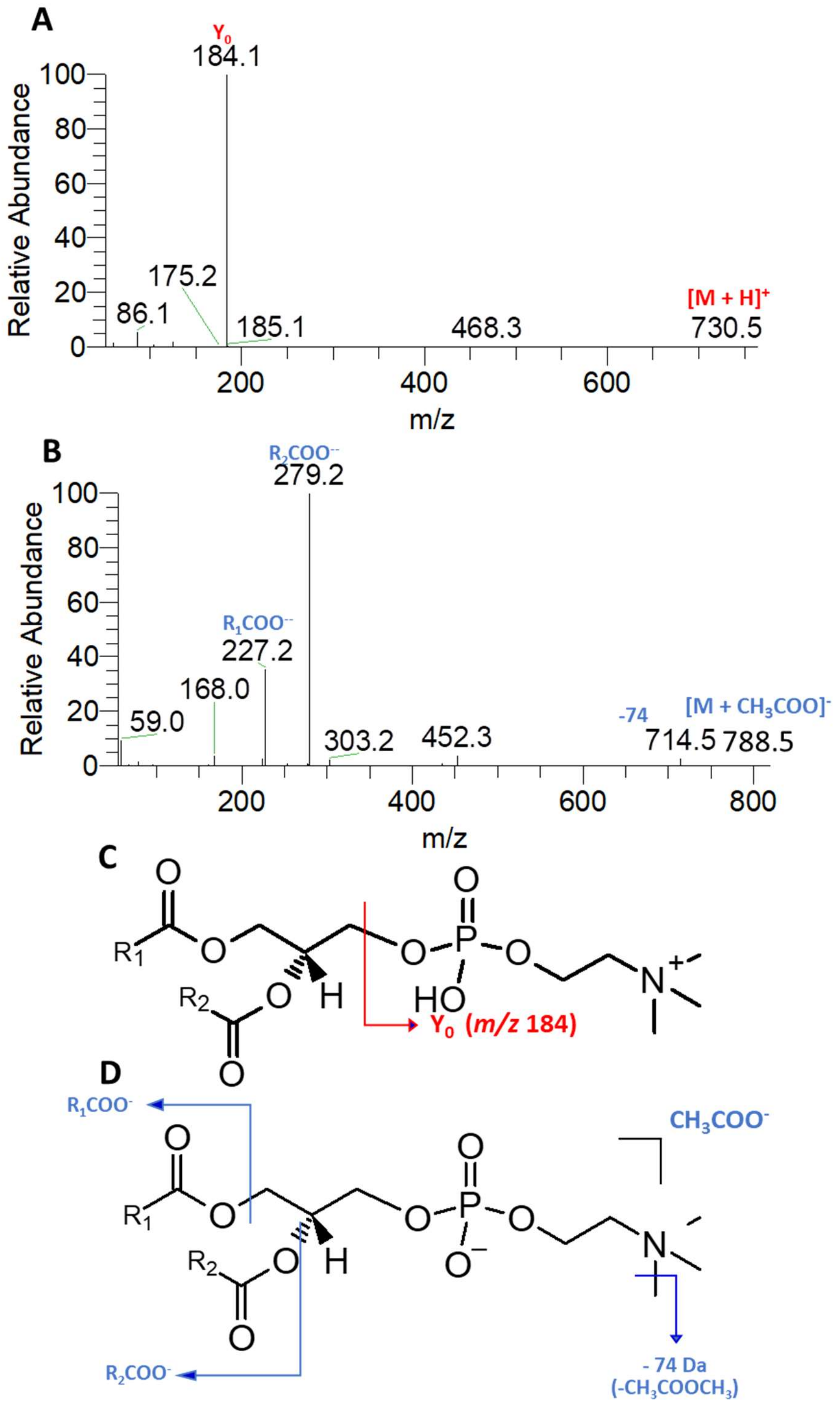

Supplementary Figure 5: Species of phosphatidylcholine (PC) confirmation by MS/MS. A) HILIC-ESI-MS/MS spectrum of PC (32:2; 14:0/18:2) corresponding to $[M + H]^+$ ion at *m/z* 730.5 in positive mode. B) HILIC-ESI-MS/MS spectrum of PC (32:2; 14:0/18:2) as $[M + CH_3COO]^-$ ion at *m/z* 788.5 in negative mode. C) Confirmation of phospholipid class was achieved by the identification of the ion at *m/z* 184.1 corresponding to the phosphocholine cation in the positive mode. D) Fatty acid components were confirmed by the identification of product ions corresponding to the fatty acyl chains as $[RCOO]^-$ in negative mode. For LPC confirmation the same principle applies, with the exception that only one product ion corresponding to a fatty acid is detected.

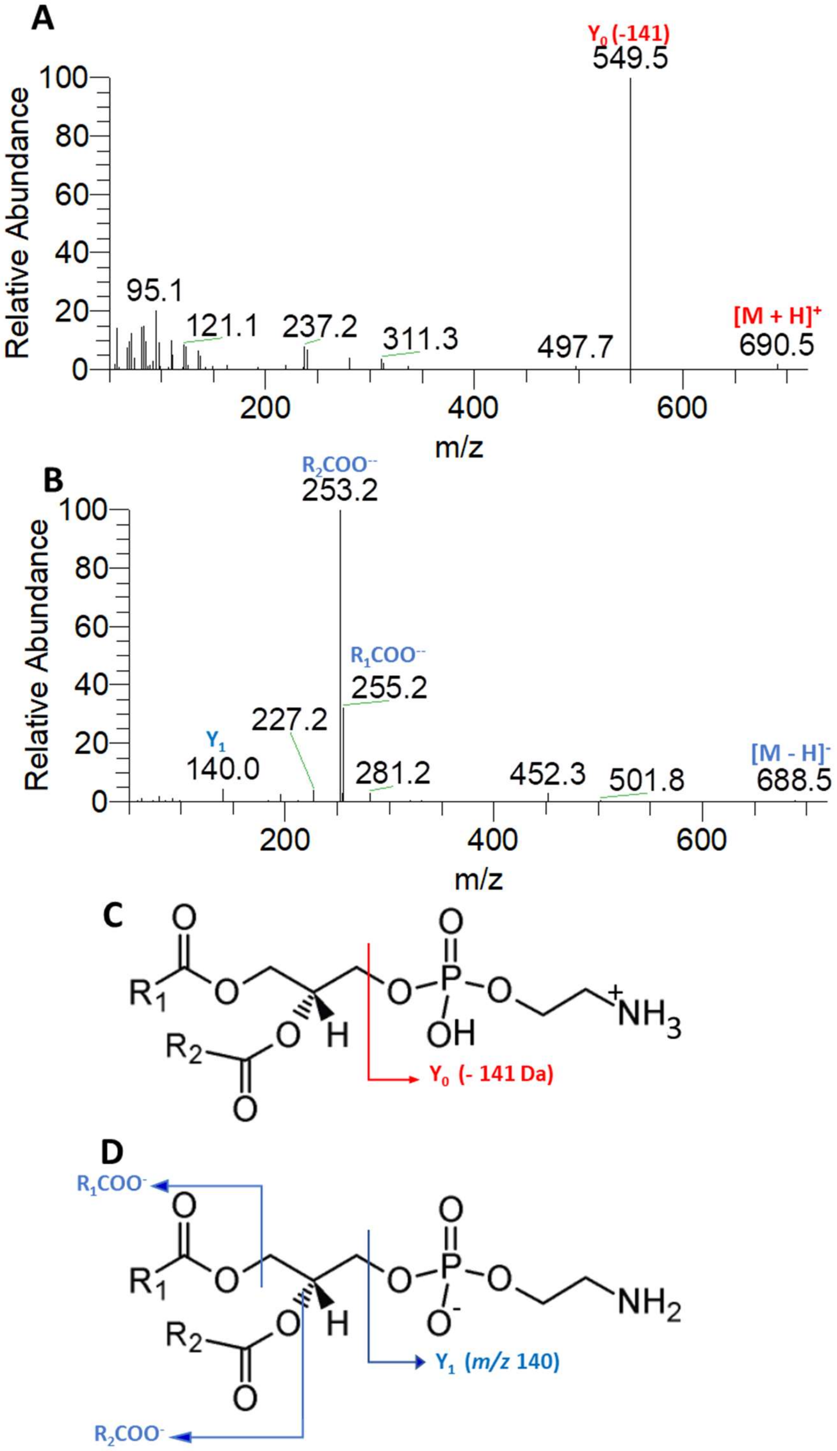

Supplementary Figure 6: Species of phosphatidylcholine (PE) confirmation by MS/MS. A) HILIC-ESI-MS spectrum of PE (32:1; 16:0/16:1) corresponding to $[M + H]^+$ ion at *m/z* 690.5 in positive mode. B) HILIC-ESI-MS/MS spectrum of PE (32:1; 16:0/16:1) as $[M - H]^-$ ion at *m/z* 688.5 in negative mode. C) Confirmation of phospholipid class was achieved by the identification of the neutral loss of -141 Da corresponding to phosphatoethanolamine from the polar head group of PE, in positive mode. D) Fatty acid components were confirmed by the identification of product ions corresponding to the fatty acyl chains as $[RCOO]^-$ in negative mode. For LPE confirmation the same principle applies, with the exception that only one product ion corresponding to a fatty acid is detected.

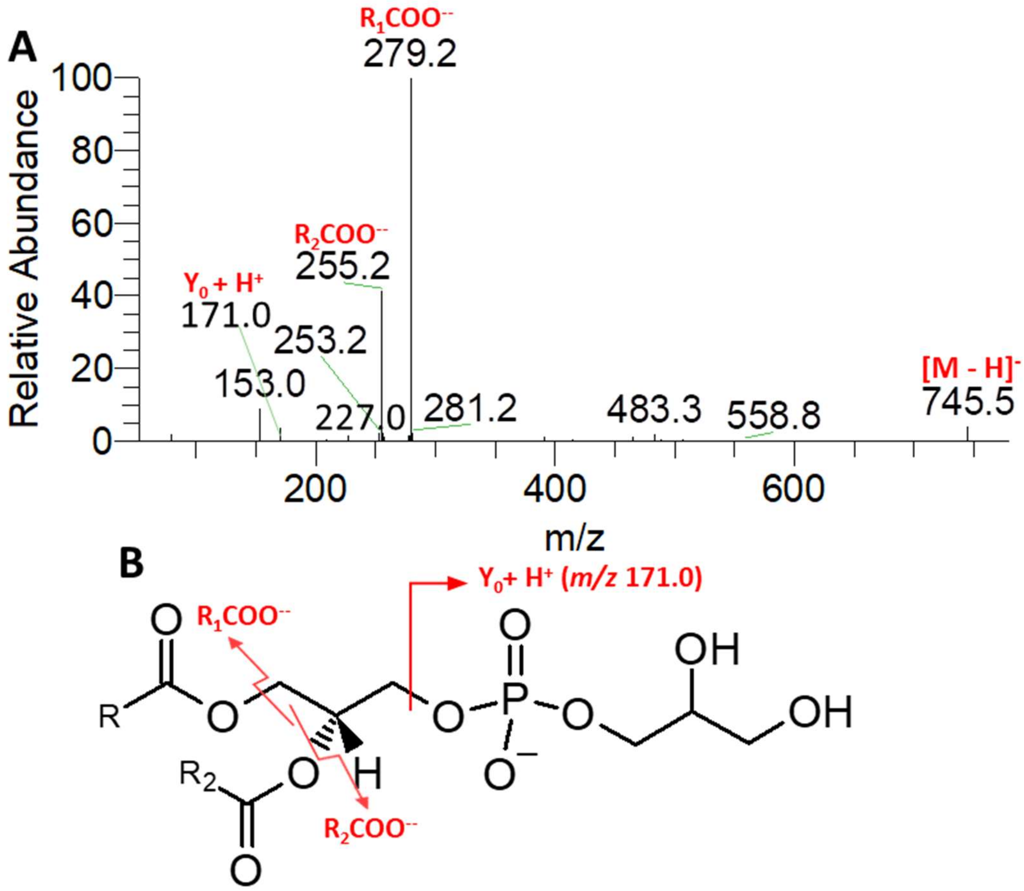

Supplementary Figure 7: Species of phosphatidylglycerol (PG) confirmation by MS/MS. A) HILIC-ESI-MS/MS spectrum of PG (34:2; 16:0 /18:2) corresponding to $[M - H]^-$ ion at *m/z* 745.5. B) Confirmation of phospholipid class was achieved by the identification of the ion at *m/z* 171.0, corresponding to the glycerol phosphate anion. Fatty acid components were confirmed by the identification of product ions corresponding to the fatty acyl chains as $[RCOO]^-$. For LPG confirmation the same principle applies, with the exception that only one product ion corresponding to a fatty acid is detected.

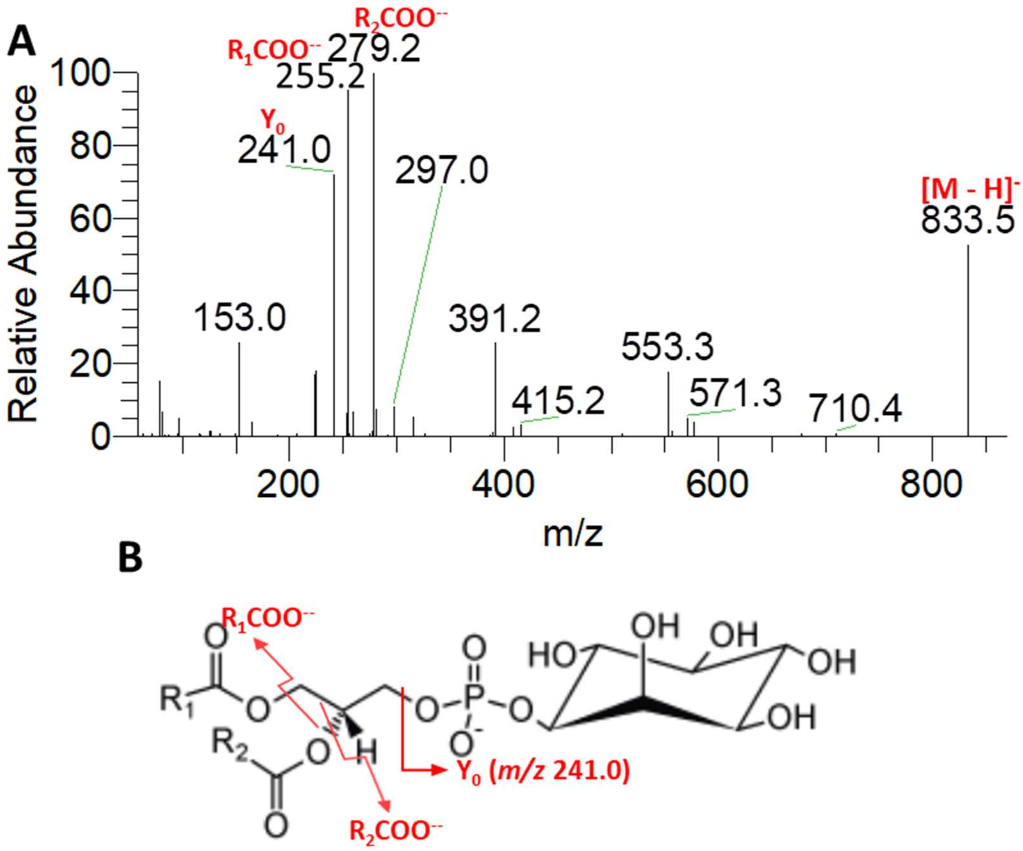

Supplementary Figure 8: Species of phosphatidylglycerol (PI) confirmation by MS/MS. a) A) HILIC-ESI-MS/MS spectrum of PI (34:2; 16:0/18:2) corresponding to $[M - H]^-$ ion at *m/z* 833.5. B) Confirmation of phospholipid class was achieved by the identification of the product ion at *m/z* 241.0 corresponding to the inositol head group. Fatty acid components were confirmed by the identification of product ions corresponding to the fatty acyl chains as $[RCOO]^-$.

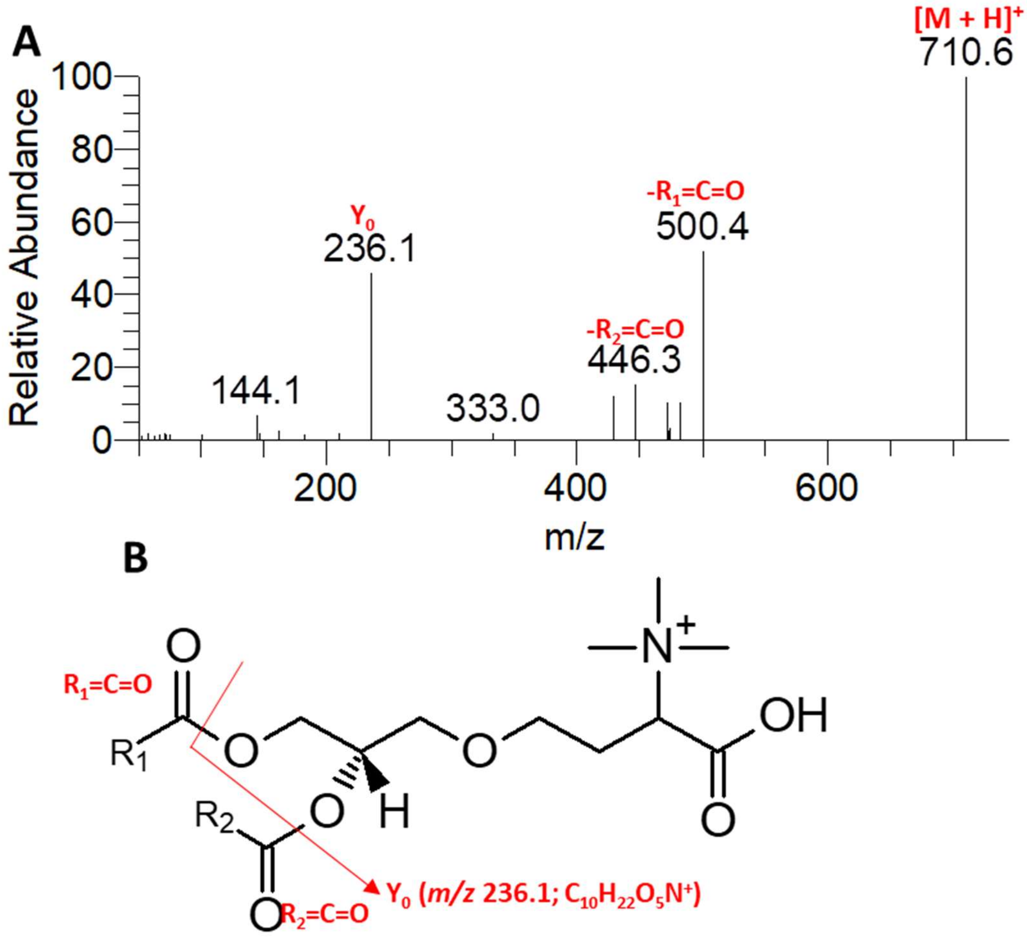

Supplementary Figure 9: Species of diacylglyceryl-N,N,N-trimethyl homoserine (DGTS) molecular species confirmation by MS/MS. A) HILIC-ESI-MS/MS spectrum of DGTS (32:1; 14:0/18:1) corresponding to $[M + H]^+$ ion at *m/z* 710.6. B) Confirmation of lipid class was achieved by the identification of the product ion at *m/z* 236.1, corresponding to $C_{10}H_{22}O_5N^+$ from the polar head group. Fatty acid components were confirmed by the identification of neutral losses of fatty acyl chains as ketenes (R=C=O).

Supplementary Table S1

Total molecular species identified by mass accuracy HILIC–LC–MS and MS/MS analyses in Saccharina latissima samples.
C represents the total number of carbon atoms and N the total number of double bonds on the fatty acyl chains.

Lipid species (C:N)	Calculated *m/z*	Observed *m/z*	Error (ppm)	Fatty acyl chains (C:N)	Formula
			**MGDG identified as $[M+NH_4]^+$**		
DGDG (32:7)	896.5371	896.5366	-0.6113	*a*	C47H78O15N
DGDG (32:4)	902.5841	902.5874	3.6295	*a*	C47H84O15N
DGDG (32:3)	904.5997	904.5964	-3.6020	*a*	C47H86O15N
DGDG (32:2)	906.6154	906.6146	-0.8671	*a*	C47H88O15N
DGDG (32:1)	908.6310	908.6293	-1.9236	*a*	C47H90O15N
DGDG (34:7)	924.5684	924.5668	-1.7156	*a*	C49H82O15N
DGDG (34:4)	930.6154	930.6178	2.5810	*a*	C49H88O15N
DGDG (34:2)	934.6467	934.6455	-1.3024	*a*	C49H92O15N
DGDG (34:1)	936.6623	936.6608	-1.5523	(16:0/18:1)	C49H94O15N
DGDG (36:8)	950.5841	950.5846	0.4933	*a*	C51H84O15N
DGDG (36:7)	952.5997	952.5977	-2.1033	*a*	C51H86O15N
DGDG (36:5)	956.6310	956.6317	0.7165	*a*	C51H90O15N
DGDG (38:9)	976.5997	976.5998	0.1155	(18:4/20:5)	C53H86O15N
DGDG (38:8)	978.6154	978.6115	-3.9892	*a*	C53H88O15N
DGDG (38:7)	980.6310	980.6325	1.4790	*a*	C53H90O15N
			**DGMG identified as $[M+NH_4]^+$**		
DGMG (16:1)*	670.4014	670.3997	-2.5003	*a*	C31H60NO14
DGMG (16:0)*	672.4170	672.4175	0.7410	*a*	C31H62NO14
DGMG (18:4)	692.3857	692.3862	0.6681	(18:4)	C33H58NO14
DGMG (18:3)	694.4014	694.4013	-0.0971	*a*	C33H60NO14
DGMG (18:2)*#	696.4170	696.4183	1.9257	*a*	C33H62NO14
DGMG (18:1)*#	698.4327	698.4320	-0.9870	*a*	C33H64NO14

**MGDG identified as $[M+NH_4]^+$**					
MGDG (32:3)	742.5464	742.5466	0.2518	*a*	C41H76NO10
MGDG (32:2)	744.5620	744.5600	-2.6758	(14:0/18:2) and (16:1/16:1)	C41H78NO10
MGDG (32:1)	746.5777	746.5767	-1.3524	(14:0/18:1) and (16:0/16:1)	C41H80NO10
MGDG (34:3)	770.5782	770.5764	-2.3832	*a*	C43H80NO10
MGDG (34:2)	772.5933	772.5908	-3.1885	*a*	C43H82NO10
MGDG (34:1)	774.6090	774.6086	-0.4667	(16:0/18:1)	C43H84NO10
MGDG (36:9)	786.5156	786.5126	-3.8043	*a*	C45H72NO10
MGDG (36:8)	788.5313	788.5297	-1.9940	(18:4/18:4)	C45H74NO10
MGDG (36:7)	790.5469	790.5463	-0.7882	*a*	C45H76NO10
MGDG (36:5)	794.5782	794.5755	-3.3385	*a*	C45H80NO10
MGDG (38:9)	814.5469	814.5439	-3.6868	(18:4/20:5)	C47H76NO10
MGDG (38:4)*	824.6251	824.6250	-0.0769	*a*	C47H86NO10
**MGMG identified as $[M+NH_4]^+$**					
MGMG (14:0)	482.3329	482.3321	-1.7473	*a*	C23H48NO9
MGMG (16:4)	502.3016	502.3010	-1.2557	*a*	C25H44NO9
MGMG (16:2)	506.3329	506.3319	-2.0216	*a*	C25H48NO9
MGMG (16:1)	508.3486	508.3481	-0.9492	*a*	C25H50NO9
MGMG (18:4)	530.3329	530.3327	-0.3814	(18:4)	C27H48NO9
MGMG (18:3)	532.3486	532.3498	2.3866	(18:3)	C27H50NO9
MGMG (18:2)	534.3642	534.3635	-1.3516	(18:2)	C27H52NO9
MGMG (18:1)	536.3799	536.3786	-2.3869	(18:1)	C27H54NO9
**SQDG identified as $[M-H]^-$**					
SQDG (28:0)	737.4510	737.4518	1.1498	(14:0/14:0)	C37H69O12S
SQDG (30:2)	761.4510	761.4505	-0.5800	*a*	C39H69O12S
SQDG (30:1)	763.4666	763.4658	-1.0406	(14:0/16:1)	C39H71O12S
SQDG (30:0)	765.4823	765.4815	-1.0573	(14:0/16:0)	C39H73O12S
SQDG (32:4)	785.4510	785.4511	0.1238	(14:0/18:4)	C41H69O12S

SQDG (32:3)	787.4666	787.4668	0.1925	(14:0/18:3)	C41H71O12S
SQDG (32:2)	789.4823	789.4825	0.2711	(14:0/18:2)	C41H73O12S
SQDG (32:1)	791.4979	791.4989	1.1745	(16:0/16:1) and (14:0/18:1)	C41H75O12S
SQDG (32:0)	793.5136	793.5124	-1.5406	*a*	C41H77O12S
SQDG (34:5)	811.4666	811.4679	1.5607	*a*	C43H71O12S
SQDG (34:4)	813.4823	813.4830	0.8709	(14:0/20:4), (16:0/18:4) and (16:1/18:3)	C43H73O12S
SQDG (34:3)	815.4979	815.4981	0.2318	(16:0/18:3) and (16:1/18:2)	C43H75O12S
SQDG (34:2)	817.5136	817.5136	-0.0146	(16:0/18:2)	C43H77O12S
SQDG (34:1)	819.5292	819.5266	-3.1529	(16:0/18:1)	C43H79O12S
SQDG (36:7)	835.4666	835.4673	0.7853	*a*	C45H71O12S
SQDG (36:6)	837.4823	837.4817	-0.7186	(18:3/18:3)	C45H73O12S
SQDG (36:5)	839.4979	839.4977	-0.2110	(16:0/20:5) and (18:2/18:3)	C45H75O12S
SQDG (36:3)	843.5292	843.5291	-0.1921	*a*	C45H79O12S
SQDG (36:2)	845.5449	845.5441	-0.9407	*a*	C45H81O12S
SQDG (36:1)*	847.5605	847.5589	-1.8705	(16:0/20:1)	C45H83O12S
SQDG (36:0)	849.5762	849.5767	0.6709	(16:0/20:0)	C45H85O12S
SQDG (38:9)	859.4666	859.4665	-0.1030	(18:4/20:5)	C47H71O12S
SQDG (38:8)	861.4823	861.4797	-3.0366	*a*	C47H73O12S
SQDG (38:1)*#	875.5918	875.5907	-1.2334	(16:0/22:1)	C47H87O12S
SQDG (38:0)*	877.6075	877.6055	-2.2392	(16:0/22:0)	C47H89O12S
SQDG (40:8)	889.5136	889.5105	-3.4831	*a*	C49H77O12S
SQDG (40:2)*#	901.6075	901.6083	0.9408	(18:1/22:1)	C49H89O12S
SQDG (40:0)	905.6388	905.6369	-2.0364	*a*	C49H93O12S
**SQMG identified as [M-H]$^-$**					
SQMG (14:0)	527.2526	527.2529	0.6267	*a*	C23H43O11S
SQMG (16:1)	553.2683	553.2690	1.3771	*a*	C25H45O11S
SQMG (16:0)	555.2839	555.2842	0.4308	(16:0)	C25H47O11S
SQMG (18:3)*#	577.2683	577.2688	0.9194	*a*	C27H45O11S
SQMG (18:2)	579.2839	579.2845	0.9656	*a*	C27H47O11S

SQMG (18:1)	581.2996	581.3008	2.0921	*a*	C27H49O11S
SQMG (24:1)*#	665.3935	665.3948	1.9463	(24:0)	C33H61O11S
SQMG (24:0)	667.4091	667.4108	2.4886	*a*	C33H63O11S
			**LPA identified as [M-H]⁻**		
LPA (20:4)	457.235517	457.235004	-1.1213	(20:4)	C23H38O7P
			**PA identified as [M-H]⁻**		
PA (30:1)	617.418233	617.419042	1.3110	*a*	C33H62O8P
PA (34:8)	659.371283	659.371299	0.0240	*a*	C37H56O8P
PA (36:5)	693.449533	693.450256	1.0431	(14:0/22:5) and (16:0/20:5)	C39H66O8P
PA (40:8)	743.465183	743.465759	0.7751	(20:4)	C43H68O8P
			**LPC identified as [M+H]⁺**		
LPC (14:0)	468.3090	468.3088	-0.4433	(14:0)	C22H47NO7P
LPC (16:2)	492.3090	492.3088	-0.4944	*a*	C24H47NO7P
LPC (16:1)	494.3247	494.3234	-2.6148	(16:1)	C24H49NO7P
LPC (16:0)	496.3403	496.3392	-2.2283	(16:0)	C24H51NO7P
LPC (18:4)	516.3090	516.3077	-2.6114	(18:4)	C26H47NO7P
LPC (18:3)	518.3247	518.3234	-2.4698	(18:3)	C26H49NO7P
LPC (18:2)	520.3403	520.3388	-2.9173	(18:2)	C26H51NO7P
LPC (18:1)	522.3560	522.3549	-1.9821	(18:1)	C26H53NO7P
LPC (18:0)	524.3716	524.3700	-3.1531	(18:0)	C26H55NO7P
LPC (20:5)	542.3247	542.3235	-2.1883	(20:5)	C28H49NO7P
LPC (20:4)	544.3403	544.3396	-1.2974	(20:4)	C28H51NO7P
LPC (20:3)	546.3560	546.3540	-3.6590	(20:3)	C28H53NO7P
LPC (20:2)	548.3716	548.3716	-0.0179	(20:2)	C28H55NO7P
LPC (20:1)	550.3873	550.3873	0.0385	(20:1)	C28H57NO7P
LPC (20:0)*	552.4029	552.4022	-1.2421	*a*	C28H59NO7P
LPC (22:6)	568.3403	568.3384	-3.3833	(22:6)	C30H51NO7P
LPC (22:5)	570.3560	570.3544	-2.6928	*a*	C30H53NO7P

**PC identified as [M+H]$^+$**					
PC (30:3)	700.4917	700.4897	-2.8974	*a*	C38H71NO8P
PC (30:1)	704.5230	704.5234	0.4976	(14:0/16:1)	C38H75NO8P
PC (30:0)	706.5387	706.5376	-1.4685	(14:0/16:0)	C38H77NO8P
PC (32:4)	726.5074	726.5075	0.1753	(14:0/18:4)	C40H73NO8P
PC (32:3)	728.5230	728.5233	0.3779	(14:0/18:3)	C40H75NO8P
PC (32:2)	730.5387	730.5372	-2.0580	(14:0/18:2), (16:0/16:2) and (16:1/16:1)	C40H77NO8P
PC (32:0)	734.5700	734.5676	-3.2171	*a*	C40H81NO8P
PC (34:7)	748.4917	748.4892	-3.4119	*a*	C42H71NO8P
PC (34:4)	754.5387	754.5380	-0.8439	(14:0/20:4), (16:0/18:4) and (16:1/18:3)	C42H77NO8P
PC (34:3)	756.5543	756.5525	-2.3605	(14:0/20:2), (16:1/18:1) and (16:0/18:2)	C42H79NO8P
PC (34:2)	758.5700	758.5674	-3.3433	*a*	C42H81NO8P
PC (36:7)	776.5230	776.5200	-3.9476	*a*	C44H75NO8P
PC (36:6)	778.5387	778.5362	-3.2116	(16:1/20:5) and (16:2/20:4)	C44H77NO8P
PC (36:4)	782.5700	782.5686	-1.7446	(16:0/20:4), (18:1/18:3) and (18:2/18:2)	C44H81NO8P
PC (36:3)	784.5856	784.5824	-4.0656	(16:0/20:3) and (18:1/18:2)	C44H83NO8P
PC (36:2)	786.6013	786.6008	-0.6043	(16:0/20:2), (18:0/18:2) and (18:1/18:1)	C44H85NO8P
PC (38:9)	800.5230	800.5213	-2.1239	(18:4/20:5)	C46H75NO8P
PC (38:8)	802.5387	802.5367	-2.5103	(18:4/20:4) and (18:3/20:5)	C46H77NO8P
PC (38:7)	804.5543	804.5528	-1.8454	(16:1/20:6), (18:2/20:5) and (18:3/20:4)	C46H79NO8P
PC (38:6)	806.5700	806.5695	-0.6143	(16:0/22:6), (18:1/20:5), (18:2/20:4) and (18:3/20:3)	C46H81NO8P
PC (38:5)	808.5856	808.5846	-1.2904	*a*	C46H83NO8P
PC (38:4)	810.6013	810.5986	-3.3652	(18:0/20:4)	C46H85NO8P
PC (38:3)	812.6169	812.6149	-2.5397	*a*	C46H87NO8P
PC (38:2)	814.6326	814.6312	-1.6598	*a*	C46H89NO8P
PC (40:10)	826.5387	826.5391	0.4702	(20:5/20:5)	C48H77NO8P
PC (40:9)	828.5543	828.5541	-0.2425	(20:4/20:5)	C48H79NO8P
PC (40:8)	830.5700	830.5672	-3.3474	(20:3/20:5) and (20:4/20:4)	C48H81NO8P

				(18:0/ 22:6), (20:1/20:5), (20:2/20:4) and	
PC (40:6)	834.6013	834.6015	0.2410	(20:3/20:3)	C48H85NO8P
PC (40:4)	838.6326	838.6312	-1.6550	*a*	C48H89NO8P
PC (40:2)*#	842.6639	842.6646	0.8878	*a*	C48H93NO8P
PC (42:11)	852.5543	852.5542	-0.1784	(20:5/22:6)	C50H79NO8P
PC (42:7)*	860.6169	860.6145	-2.8733	(20:1/22:6)	C50H87NO8P
PC (42:6)*	862.6326	862.6309	-2.0022	(20:5/22:1)	C50H89NO8P
PC (42:2)*#	870.6952	870.6943	-1.0599	*a*	C50H97NO8P
PC (44:12)	878.5700	878.5687	-1.4440	(22:6/22:6)	C52H81NO8P
PC (44:10)	882.6013	882.6015	0.2018	*a*	C52H85NO8P
PC (44:6)*	890.6639	890.6649	1.1141	(20:5/24:1)	C52H93NO8P
**LPE identified as $[M-H]^-$**					
LPE(16:0)	452.2777	452.2784	1.5962	(16:0)	C21H43NO7P
LPE(18:4)*	472.2464	472.2469	1.0261	*a*	C23H39NO7P
LPE(18:3)*	474.2621	474.2631	2.2096	(18:3)	C23H41NO7P
LPE(18:2)*	476.2777	476.2782	1.1039	*a*	C23H43NO7P
LPE(18:1)	478.2934	478.2940	1.4121	(18:1)	C23H45NO7P
LPE(18:0)	480.3090	480.3098	1.5996	(18:0)	C23H47NO7P
LPE(20:5)	498.2621	498.2627	1.3046	*a*	C25H41NO7P
LPE(20:4)	500.2777	500.2788	2.0747	*a*	C25H43NO7P
LPE(20:1)	506.3247	506.3245	-0.3705	*a*	C25H49NO7P
LPE(22:6)	524.2777	524.2778	0.2388	(22:6)	C27H43NO7P
**PE identified as $[M-H]^-$**					
PE(30:2)	658.4448	658.4462	2.0834	*a*	C35H65NO8P
PE(30:1)	660.4604	660.4608	0.5390	(14:0/16:1) and (14:1/16:0)	C35H67NO8P
PE(30:0)	662.4761	662.4754	-0.9918	*a*	C35H69NO8P
PE(32:3)$	684.4604	684.4595	-1.3793	*a*	C37H67NO8P
PE(32:2)	686.4761	686.4763	0.2643	(14:0/18:2), (16:0/16:2) and (16:1/16:1)	C37H69O8NP
PE(32:1)	688.4917	688.4921	0.5020	(14:0/18:1) and (16:0/16:1)	C37H71NO8P

PE(34:5)	708.4604	708.4609	0.6411	(14:0/20:5)	C39H67NO8P
PE(34:4)	710.4761	710.4756	-0.6662	(14:0/20:4), (16:0/18:4) and (16:1/18:3)	C39H69NO8P
PE(34:3)	712.4917	712.4909	-1.1966	*a*	C39H71O8NP
PE(34:2)	714.5074	714.5059	-2.0655	(16:0/18:2) and (16:1/18:1)	C39H73NO8P
PE(34:1)	716.5230	716.5222	-1.1654	(16:0/18:1) and (16:1/18:0)	C39H75NO8P
PE(36:6)	734.4761	734.4770	1.3134	*a*	C41H69NO8P
PE(36:5)	736.4917	736.4925	0.9778	(16:0/20:5), (16:1/20:4) and (18:2/18:3)	C41H71O8NP
PE(38:5)	764.5230	764.5220	-1.2719	*a*	C43H75NO8P
PE(38:4)	766.5390	766.5388	-0.2694	*a*	C43H77O8NP
PE(40:9)	784.4917	784.4896	-2.7649	(20:4/20:5)	C45H71O8NP
PE(40:8)	786.5074	786.5073	-0.0709	(20:4/20:4)	C45H73O8NP
PE(40:5)	792.5543	792.5522	-2.6922	*a*	C45H79O8NP
PE(40:4)	794.5700	794.5712	1.5672	(20:0/20:4)	C45H81NO8P
**LPG identified as $[M-H]^-$**					
LPG(16:1)	481.2566	481.2570	0.6949	(16:1)	C22H42O9P
LPG(16:0)	483.2723	483.2725	0.4399	(16:0)	C22H44O9P
LPG(18:2)	507.2723	507.2725	0.3288	(18:2)	C24H44O9P
LPG(18:1)	509.2879	509.2880	0.0811	(18:1)	C24H46O9P
LPG(22:5)	557.2879	557.2865	-2.6122	*a*	C28H46O9P
**PG identified as $[M-H]^-$**					
PG(30:1)	691.4550	691.4556	0.8515	(14:0/16:1) and (14:1/16:0)	C36H68O10P
PG(30:0)	693.4707	693.4711	0.6441	(14:0/16:0) and (15:0/15:0)	C36H70O10P
PG(32:2)	717.4707	717.4719	1.6547	(16:1/16:1)	C38H70O10P
PG(32:1)	719.4863	719.4865	0.3219	(14:0/18:1) and (16:0/16:1)	C38H72O10P
PG(34:5)	739.4550	739.4543	-0.9949	(14:0/20:5), (16:3/18:2) and (16:1/18:4)	C40H68O10P
PG(34:4)	741.4707	741.4710	0.3663	(14:0/20:4), (16:0/18:4), (16:1/18:3) and (16:2/18:2)	C40H70O10P
PG(34:3)	743.4863	743.4871	1.0386	(16:0/18:3) and (16:1/18:2)	C40H72O10P
PG(34:2)	745.5020	745.5022	0.2291	(16:0/18:2) and (16:1/18:1)	C40H74O10P
PG(34:1)	747.5176	747.5177	0.1939	(16:0/18:1)	C40H76O10P

PG(35:2)#	759.5182	759.5158	-3.0970	*a*	C41H76O10P
PG(36:4)	769.5020	769.5003	-2.1708	(18:2/18:2)	C42H74O10P
PG(36:3)	771.5176	771.5178	0.2831	*a*	C42H76O10P
PG(36:2)	773.5333	773.5331	-0.2410	(16:0/20:2), (16:1/20:1), (18:0/18:2) and (18:1/18:1)	C42H78O10P
PG(36:1)	775.5489	775.5464	-3.1777	(16:0/20:1) and (18:0/18:1)	C42H80O10P
PG(38:5)	795.5176	795.5162	-1.8215	*a*	C44H76O10P
PG(40:6)	821.5333	821.5342	1.0438	*a*	C46H78O10P
**LPI identified as $[M\text{-}H]^-$**					
LPI(22:0)	655.3822	655.3839	2.5300	*a*	C31H60O12P
LPI(24:0)	683.4135	683.4150	2.1589	*a*	C33H64O12P
**PI identified as $[M\text{-}H]^-$**					
PI(30:1)	779.4711	779.4688	-2.9966	*a*	C39H72O13P
PI(32:2)	805.4867	805.4836	-3.8841	(14:0/18:2), (14:1/18:1) and (16:1/16:1)	C41H74O13P
PI(32:1)	807.5024	807.5038	1.7066	(14:0/18:1) and (16:0/16:1)	C41H76O13P
PI(34:7)	823.4398	823.4380	-2.1204	*a*	C43H68O13P
PI(33:0)	823.5337	823.5343	0.8113	*a*	C42H80O13P
PI(34:4)	829.4867	829.4833	-4.1250	(14:0/20:4) and (16:0/18:4)	C43H74O13P
PI(34:3)	831.5024	831.5023	-0.1584	*a*	C43H76O13P
PI(34:2)	833.5180	833.5147	-3.8997	(16:0/18:2) and (16:1/18:1)	C43H78O13P
PI(34:1)	835.5337	835.5336	-0.1588	(16:0/18:1)	C43H80O13P
PI(36:4)	857.5180	857.5158	-2.5820	(16:0/20:4), (18:1/18:3) and (18:2/18:2)	C45H78O13P
PI(36:2)	861.5493	861.5478	-1.7520	*a*	C45H82O13P
PI(38:10)	873.4554	873.4519	-4.0657	*a*	C47H70O13P
PI(38:6)	881.5180	881.5187	0.8464	*a*	C47H78O13P
PI(38:5)*	883.5337	883.5334	-0.3379	*a*	C47H80O13P
PI(38:4)*	885.5493	885.5486	-0.8465	*a*	C47H82O13P
PI(40:6)*	909.5493	909.5488	-0.5601	*a*	C49H82O13P
**MGTS identified as $[M+H]^+$**					

MGTS(14:0)	446.3482	446.3474	-1.7985	*a*	C24H48O6N
MGTS(16:0)	474.3795	474.3798	0.6703	*a*	C26H52O6N
**DGTS identified as [M+H]$^+$**					
DGTS(30:1)	682.5622	682.5625	0.4872	(14:0/16:1)	C40H76O7N
DGTS(32:2)	708.5778	708.5782	0.5142	(16:1/16:1)	C42H78O7N
DGTS(32:1)	710.5935	710.5936	0.1428	(14:0/18:1) and (16:0/16:1)	C42H80O7N
DGTS(32:0)	712.6091	712.6060	-4.2906	*a*	C42H82O7N
DGTS(34:4)	732.5778	732.5754	-3.2167	*a*	C44H78O7N
DGTS(34:3)	734.5935	734.5921	-1.8653	(16:0/18:3)	C44H80O7N
DGTS(34:2)	736.6091	736.6093	0.2710	(16:0/18:2) and (16:1/18:1)	C44H82O7N
DGTS(34:1)	738.6248	738.6233	-2.0760	(16:0/18:1)	C44H84O7N
DGTS(36:5)	758.5935	758.5936	0.1440	*a*	C46H80O7N
DGTS(36:3)	762.6248	762.6246	-0.2391	(18:1/18:2)	C46H84O7N
DGTS(38:5)*	786.6248	786.6224	-3.1078	*a*	C48H84O7N

a: no MS/MS spectra; $: not present in FR; *: not present in NO; #: not present in UK.
